# Cadmium exposure accelerates cognitive deficits and Alzheimer's disease progression in 5xFAD mice

**DOI:** 10.1002/alz70855_103810

**Published:** 2025-12-24

**Authors:** Steven G Dixon, Maxwell Cook, Steven L Carroll, Gavin Y Wang

**Affiliations:** ^1^ Medical University of South Carolina, Charleston, SC, USA

## Abstract

**Background:**

Epidemiological studies have identified an association between the exposure to neurotoxic heavy metals such as cadmium (Cd) and an increased risk for Alzheimer's disease (AD). However, the cellular and molecular mechanisms by which Cd or lead (Pb) exposure affects AD pathogenesis and progression are largely unknown. The goal of this study was to determine how Cd and Pb exposure impact cognitive function and AD progression using the 5xFAD transgenic mouse model of AD.

**Methods:**

Juvenile (5‐weeks old) 5xFAD mice were challenged with 0.2% Pb or 0.002% Cd in drinking water for 6 weeks to model chronic environmental exposures. Y‐maze tests were performed to assess spatial memory and learning ability. The Open Field Maze test was employed to examine anxiety‐like behaviors. Senescent cells were determined using senescence‐associated β‐galactosidase staining. Quantitative RT‐PCR assays were performed to measure expression levels of senescence markers and senescence‐associated secretory phenotype (SASP) related cytokines. Immunofluorescence and confocal microscopy were used to analyze senescent cells and neuroinflammation markers in brain tissues.

**Results:**

Cd and Pb both can accelerate the pace of amyloid beta (Aβ) plaque formation and the decline of cognitive functions in 5xFAD mice. Even at a 100‐fold lower dose, chronic Cd exposure causes a greater level of Aβ deposition and cognitive deficits than Pb does, indicating that Cd is more potent than Pb in exacerbating AD progression. Mechanistically, we found that the increased expression of the p16^Ink4a^ senescence marker was more pronounced in the hippocampus of Cd‐exposed mice compared to those treated with Pb. Notably, Cd upregulates the expression of the SASP marker IL‐6 preferentially in the hippocampus over the cortex, suggesting a spatial difference in Cd‐induced increase in neuroinflammation. Furthermore, *in vitro* mechanistic studies reveal that Cd exposure induces premature senescence in human microglial cells, and senescent microglia release high levels of IL‐6, a major modulator of neuroinflammation.

**Conclusion:**

Our studies demonstrate that chronic exposure to Cd or Pb can exacerbate cognitive deficits and AD progression in 5xFAD mice. Mechanistically, we discovered that Cd exposure‐induced acceleration of AD progression was associated with increased levels of senescent cell burden and neuroinflammation in the hippocampus.

